# A Dual-Band High-Sensitivity THz Metamaterial Sensor Based on Split Metal Stacking Ring

**DOI:** 10.3390/bios12070471

**Published:** 2022-06-29

**Authors:** Xuejing Lu, Hongyi Ge, Yuying Jiang, Yuan Zhang

**Affiliations:** 1 PLA Strategic Support Force Information Engineering University, Zhengzhou 450001, China; xuejinglu2011@163.com; 2 Key Laboratory of Grain Information Processing & Control, Ministry of Education, Henan University of Technology, Zhengzhou 450001, China; gehongyi@haut.edu.cn (H.G.); jiangyuying11@163.com (Y.J.); 3 College of Information Science and Engineering, Henan University of Technology, Zhengzhou 450001, China

**Keywords:** terahertz, metamaterial, dual-band sensor, high-sensitivity, refractive index

## Abstract

Terahertz (THz)-detection technology has been proven to be an effective and rapid non-destructive detection approach in biomedicine, quality control, and safety inspection, among other applications. However, the sensitivity of such a detection method is limited due to the insufficient power of the terahertz source and the low content, or ambiguous characteristics, of the analytes to be measured. Metamaterial (MM) is an artificial structure in which periodic sub-wavelength units are arranged in a regular manner, resulting in extraordinary characteristics beyond those possessed by natural materials. It is an effective method to improve the ability of terahertz spectroscopy detection by utilizing the metamaterial as a sensor. In this paper, a dual-band, high-sensitivity THz MM sensor based on the split metal stacking ring resonator (SMSRR) is proposed. The appliance exhibited two resonances at 0.97 and 2.88 THz in the range of 0.1 to 3 THz, realizing multi-point matching between the resonance frequency and the characteristic frequency of the analytes, which was able to improve the reliability and detection sensitivity of the system. The proposed sensor has good sensing performance at both resonant frequencies and can achieve highest sensitivities of 304 GHz/RIU and 912 GHz/RIU with an appropriate thickness of the analyte. Meanwhile, the advantage of multi-point matching of the proposed sensor has been validated by distinguishing four edible oils based on their different refractive indices and demonstrating that the characteristics obtained in different resonant frequency bands are consistent. This work serves as a foundation for future research on band extension and multi-point feature matching in terahertz detection, potentially paving the way for the development of high-sensitivity THz MM sensors.

## 1. Introduction

Terahertz waves (0.1–10 THz) exist in the electromagnetic spectrum between microwave and infrared and have unique spectral properties within this band. They are non-ionizing beams with low photon energy that fail to damage biomolecules when irradiating samples [1,2,3]. Moreover, THz waves have good penetrability and can pass through non-polar substances to detect internally [4]. Furthermore, terahertz spectroscopy can be used to characterize macromolecules because the vibration and rotation patterns of molecules are mostly in this frequency range [5], allowing for the generation of a distinct “fingerprint” spectrum of many substances. Therefore, terahertz spectroscopy has potential application in the fields of biomedicine [6], security inspection [7], and quality control [8], with the unique advantage of being a fast, non-destructive and label-free detection method. Nonetheless, in practice, the sensitivity and accuracy of detection are limited by the fact that the electromagnetic response of most substances in nature to THz waves is prone to be too weak [9,10]. In the field of agricultural products, the distinction of different kinds of substances is of significance for ensuring the quality and safety of grain and oil. At present, however, it is difficult to distinguish some agricultural products using terahertz time-domain spectroscopy directly, such as edible oils. Thus, developing an effective detection method with high sensitivity has practical significance for promoting the development of a THz detection technique.

Metamaterials are compositional periodic structures of artificially engineered units with exotic optical characteristics beyond those of natural materials [11,12,13]. The electromagnetic response of the incident wave can be adjusted by rationally designing the unit structure to enhance the interaction between the detected wave and the measured substance on the surface of the metamaterial, which will be more obvious when the resonant frequency of the metamaterial is consistent with the characteristic frequency of the measured substance. Recently, the maturation of the micro/nano fabrication has resulted in the rapid development of metamaterials, and terahertz metamaterial sensors are becoming an emerging research hotspot due to their good penetration and ability to characterize biological macromolecules of terahertz waves [14,15,16]. The sensing principle of terahertz metamaterial is that changes in the surrounding permittivity caused by analytes with different refractive indices will induce the variation of resonant peaks in the relevant transmission spectrum [17,18]. Thus, by placing the measured substances on the sensor’s surface, in contact with the metallic structural layer, we can measure and calculate the resonant frequency shift of the transmission or reflection spectrum relative to the characteristic spectrum of the metamaterial itself to accomplish substance identification [19,20,21]. Wang et al. [22] proposed a terahertz biosensor based on all-metal metamaterial with a maximum refractive sensitivity of 294.95 GHz/RIU and assessed this sensor’s effectiveness by detecting the bovine serum albumin. Wang et al. [23] proposed a metamaterial biosensor, which consisted of a metal elliptical split-ring resonator array with a subwavelength structure based on flexible thin film to extend the application of flexible metamaterial in the sensor field, which reached 243 GHz/RIU with a high Q-factor of 14.2. Furthermore, a dual-band terahertz metamaterial was proposed to realize detection at two electromagnetically induced transparency (EIT) resonances. The refractive index sensitivity was 280.8 GHz/RIU for sensing the refractive index in the range 1.5–2 at the first EIT resonance and 201.6 GHz/RIU for sensing the refractive index in the range 1–1.5 at the second EIT resonance [24]. With the development of high-powered stable terahertz sources, it is more relevant than ever to broaden the terahertz band availability for metamaterials, allowing the detection of the substance over a wider range of frequency bands. In addition, a metamaterial sensor with multiple resonances can realize the multi-point matching, enabling one to recognize the specific substance at multiple frequency bands simultaneously. Therefore, these kinds of broadband metamaterial sensors are becoming research hotspots.

In general, Q-factor (Q), refractive index sensitivity (S), and figure of merit (FOM) are critical parameters for evaluating the sensing performance of a terahertz metamaterial sensor [25,26]. As shown in Formula (1), the Q-factor is defined as the ratio of the resonant frequency (f) to the full width at half-maximum (FWHM) of the resonant peak [27]. The higher the Q-factor (the narrower the FWHM) is, the easier it is to distinguish the offset of the resonant peak. In other words, the sharper the resonant peak is, the more reliably the sensor’s detection shows. The refractive index sensitivity (S) reflects the relationship between the resonant frequency shift (∆f) induced by the addition of the analytes and the change of refractive index (∆n), and it can be calculated using Formula (2) [28]. The FOM value is usually used to compare sensing performance under different resonant frequency regions, which can be introduced as shown in Formula (3). A higher FOM value indicates that the metamaterial sensor has a better sensing performance. To improve sensor sensing performance, the resonant frequency and modulation depth of the metamaterial can be adjusted by modifying the unit structure or the dielectric material to enhance the electromagnetic field in a localized area, achieving a high Q-factor of resonance [29].

(1) $$Q=\frac{f}{FWHM}$$

(2) $$S=\frac{∆f}{∆n}$$

(3) $$FOM=\frac{S}{FWHM}$$

In this paper, a dual-band high-sensitivity terahertz metamaterial sensor (THz MM sensor) composed of split metal stacking ring arrays arranged on a flexible substrate of polytetrafluoroethylene (PTFE) is proposed. Such a sensor has two resonant modes within a range of 0.2–3 THz and can achieve multi-point feature matching with the characteristic frequency of the measured substance. The underlying mechanisms of both resonant modes have been investigated via the analysis of surface electric/magnetic fields and current distributions based on the electromagnetic oscillation theory. Furthermore, the sensing performance of the proposed sensor has been calculated numerically and analyzed by varying the refractive index and the thickness of the analyte layer. Finally, four edible oils were chosen to validate the detection ability of the proposed terahertz metamaterial sensor. It was shown that the THz MM sensor has a high refractive index sensitivity at both resonance modes and can achieve effective multi-point matching to the characteristics of the substance. The dual-band THz MM sensor will be fabricated using photolithography [30] and applied to distinguish various oils based on their different refractive indices. According to its high refractive index sensitivity, such a metamaterial sensor can sense the tiny changes in refractive index, which is more valuable for trace substance detection. In the field of agricultural products, the detection of pesticide residues and fungal toxins deserves to be further studied.

## 2. Structure Design and Simulation

**Figure 1:**
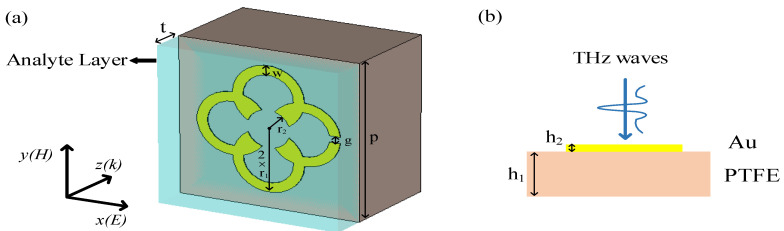
(**a**) Schematic diagram of the proposed SMSRR; (**b**) cross-sectional view of the SMSRR.

A kind of terahertz metamaterial of SMSRR is proposed, which consists of a metal layer (Au) with the electrical conductivity of $\delta =4.56\times 10^{7}$ S/m deposited onto a PTFE (with the relative permittivity of $\varepsilon_{r}=2.1$) substrate. Figure 1a,b display the schematic diagram and the cross-sectional view of the proposed THz MM sensor. Four rings of radius $r_{1}$ and width w are symmetrically superimposed, with a hollow circle with radius $r_{2}$ in the center and a split of width g on the right. These parameters were obtained from the optimization process of the design. Here, the geometrical dimensions of the unit structure in this case are p=50 μm, $r_{1}=10\;\mu m$, $r_{2}=6\;\mu m$, w=3 μm, g=2 μm, $h_{1}=30\;\mu m$, and $h_{2}=0.2\;\mu m$.

CST Studio Suite 2020 software was used to study the electromagnetic responses of the designed terahertz metamaterial and optimize the geometrical dimensions of the SMSRR structure [22]. In the simulation, the boundary condition was set as unit cell for X/Y directions to repeat the structure periodically in two directions, while it was set as open for the Z direction, which refers to the perfectly matched layer (PML) condition. As shown in Figure 1b, the terahertz transverse electromagnetic (TE) waves incident vertically on the surface of the metamaterial from the metal side, propagating in the Z direction with a horizontally polarized electric field (Ex) and a vertically polarized magnetic field (Hy).

## 3. Results and Discussion

### 3.1. Resonant Mechanism

**Figure 2:**
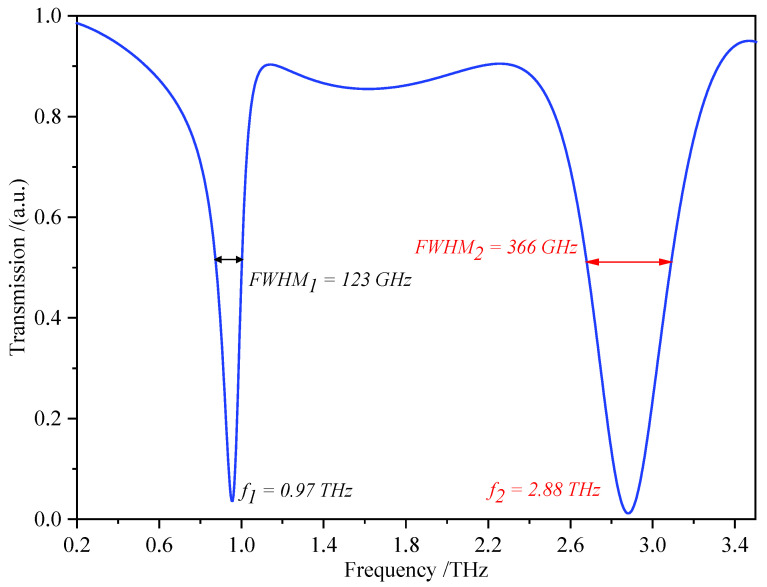
Simulated transmission spectrum of the proposed SMSRR.

Figure 2 displays the corresponding transmission spectrum of the proposed SMSRR, revealing two separate resonant peaks at the low frequency of $f_{1}=0.97$ THz and the high frequency of $f_{2}=2.88$ THz. The FWHM values of the two resonant peaks are 123 GHz and 336 GHz, as shown in the figure. According to Formula (1), the Q-factors of $f_{1}$ and $f_{2}$ are $Q(f_{1})=7.65$ and $Q(f_{2})=7.87$, respectively, with modulation depths of 96% and 99%.

**Figure 3:**
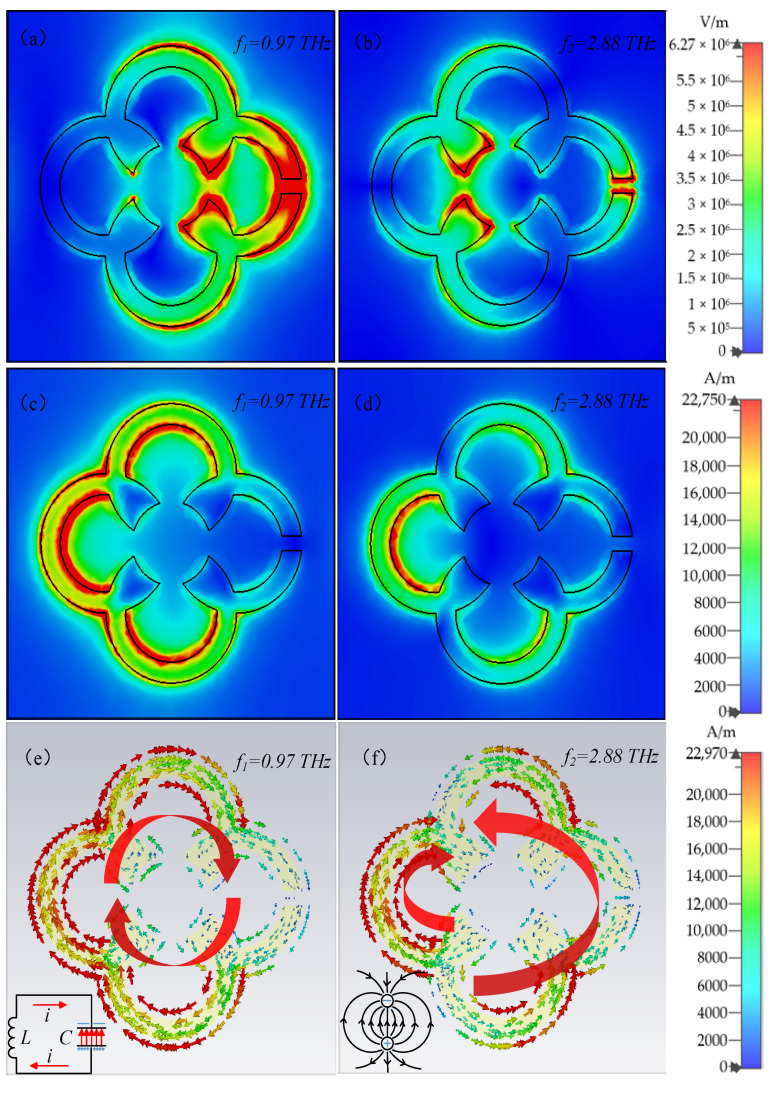
The electric field distribution at (**a**) $f_{1}$ and (**b**) $f_{2}$; the magnetic field distribution at (**c**) $f_{1}$ and (**d**) $f_{2}$; the current distribution at (**e**) $f_{1}$ (LC resonance) and (**f**) $f_{2}$ (dipole resonance).

The surface electric/magnetic fields and current distribution of the terahertz metamaterial were collected at the resonant peak frequencies to elucidate the physical mechanisms of both resonant modes. The distributions of the electric field are shown in Figure 3a,b. In the low-frequency resonant mode ($f_{1}$), the enhanced electric field was located at the gap of the unit structure, and the further away from the gap, the weaker the electric field was; in the high-frequency resonance mode ($f_{2}$), the higher induced charges concentrated at the gap and two bulges of the inner ring, forming strong electric fields to generate dipole resonance. Figure 3c,d depict the surface magnetic fields of the metamaterial structure at two resonant frequencies, which were symmetrically distributed with the electric fields.

Figure 3e exhibits the surface current distribution at $f_{1}$, showing that the current directions are consistent throughout the metamaterial structure. The induced charges concentrated at the gap move to form an equivalent LC oscillation circuit are represented in the left bottom, while the gap on the right of the unit structure can be thought of as capacitance C, and the metallic part can be assumed as inductance L, demonstrating that the resonance at $f_{1}$ is generated by LC resonance. The frequency $f_{1}$ was determined based on the well-known resonance formula: $f=1/2\pi \sqrt{LC}$. According to Figure 3f, at the higher resonant frequency $f_{2}$, the current direction on the left side of the ring is opposite to that on the right side, causing the induced charges to converge at the bulges on the left side of the inner ring, forming the electric dipole shown in the lower left of the figure. At this point, the alternating electric field drove the dipole vibration, resulting in a dipole resonance. Both resonant frequencies were determined by the equivalent capacitance and inductance related to the structure of the metal unit itself [29].

### 3.2. Sensing Performance

Using the terahertz metamaterial as a sensor for substance detection, the analytes ought to be covered on the surface of the metamaterial, which causes changes in the ambient permittivity, resulting in the resonant frequency of the metamaterial’s THz transmission spectrum shifting. The refractive index of the analyte added can be judged by measuring the offset of the resonance peak, and substances with different refractive indices can be distinguished according to this. It is of necessity to investigate the sensing performance of the dual-band THz MM sensor based on the SMSRR, aiming to improve its refractive index sensitivity and realize its practical application.

**Figure 4:**
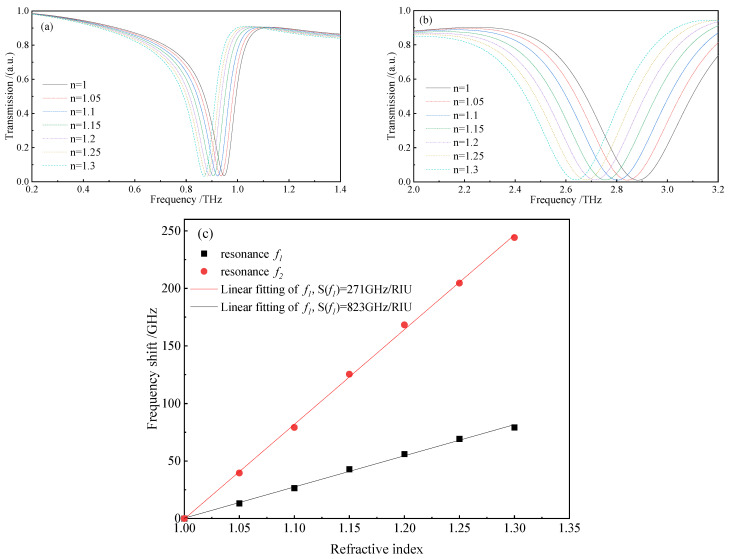
The transmission spectra of the THz MM sensor at (**a**) $f_{1}$ and (**b**) $f_{2}$ with changing ***n*** from 1.0 to 1.3; (**c**) resonance frequency shift of the sensor as a function of n and the linear fitting results.

An analyte layer with a fixed thickness of t=6 μm was placed on top of the dual-band THz MM sensor. Figure 4a,b show the terahertz transmission spectra obtained by changing the refractive index (n) of the analyte layer from 1.0 to 1.3 with a step length of 0.05 at the resonant frequencies $f_{1}$ and $f_{2}$, respectively. The resonant frequencies exhibited different degrees of redshift as the value n increased, and the substances to be measured with differing refractive indices were identified based on the value of frequency shift. Figure 4c depicts the frequency shift of the sensor as a function of the refractive index for both resonant modes. The position of the resonance peaks shifted approximately linearly with the increment of value n. The slopes of the fitting curves represent the refractive index sensitivity of the sensor, which were $S(f_{1})=271$ GHz/RIU for resonance 1 and $S(f_{2})=823$ GHz/RIU for resonance 2, respectively. Compared with the results in recent literature [31,32], the proposed dual-band THz MM sensor had higher refractive index sensitivity in both resonant frequency bands, allowing one to distinguish analytes with similar refractive indices.

**Figure 5:**
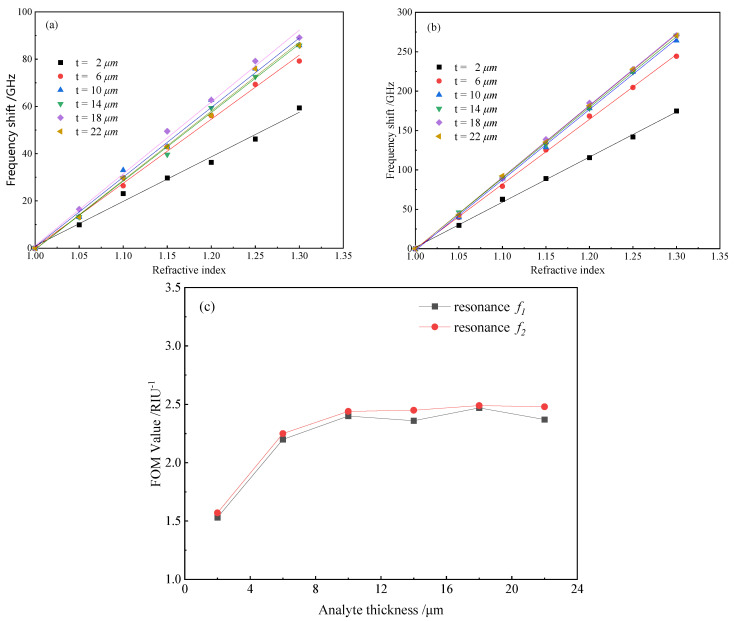
Resonant frequency shift of the sensor as a function of refractive index with different thicknesses of analyte and the linear fitting results at (**a**) $f_{1}$ and (**b**) $f_{2}$; (**c**) influence of the analyte thickness on the FOM values.

To further investigate the influence of analytes on the refractive index sensitivity of the proposed sensor, different thicknesses of the analyte layer (from 2 to 22 μm with the step length of 4 μm) were numerically simulated. Figure 5a,b exhibit the relationship between the resonant frequency shift and the refractive indices for different thickness analytes at resonant frequencies $f_{1}$ and $f_{2}$, respectively. As shown in the figures, the sensitivity increased from 188 GHz/RIU to 271 GHz/RIU for resonance 1 and from 573 GHz/RIU to 823 GHz/RIU for resonance 2 when the value of t was changed from 2 to 6 μm. Namely, as the thickness of the analyte layer increased, the refractive index sensitivity of the sensor significantly increased. However, after $t\geq 10_{}\;\mu m$, this increase in refractive index sensitivity was no longer evident and even decreased, because the analyte thickness was beyond the range of distance that electromagnetic resonance affected, which alternatively affected the sensing performance of the sensor. Thus, it was necessary to select a suitable analyte thickness during detection, which was of practical significance for improving the sensitivity of the sensor.

In order to obtain a quantitative description of the sensing performance, the refractive index sensitivity and the FOM value of the proposed THz MM sensor were calculated according to Formulae (2–3). FOM value is regularly used to compare the sensing performance of a sensor in different frequency bands. The narrower the FWHM of the resonant peak was, the less likely that the terahertz spectra of different substances overlapped, and the more accurate the detection results were. Figure 5c depicts the effect of analyte thickness on the FOM value of the sensor; since the FOM value was higher for resonance 2, the sensor performed better overall at frequency $f_{2}$ than at frequency $f_{1}$. Table 1 displays the performance parameters of the THz MM sensor at $f_{1}$ and $f_{2}$ with varying analyte thicknesses, showing that both resonant bands had better performance when t=18 μm.

Comparison with previously published metamaterial sensors using transmission spectra is essential. Li D. et. al [31] designed a sensor consisting of a metallic microsize circular ring gap patterned on the quartz substrate with a refractive index sensitivity of 29 GHz/RIU at 0.286 THz and 74 GHz/RIU at 0.850 THz, which was used in the frequency range of 0.2–1 THz. Zhu L. et. al [24] proposed a kind of metamaterial with refractive index sensitivity of 280.8 GHz/RIU at 0.89 THz and 201.6 GHz/RIU at 1.56 THz. The first resonance was suitable for sensing the refractive index of an analyte from 1 to 2, but the second resonance was only suitable for sensing the refractive index from 1 to 1.5, since further enhancement of the refractive index for the analyte raised the overall loss of metamaterial and degraded the performance of the second resonance band. In contrast, the proposed sensor in this paper has the following advantages: firstly, the two resonances are far apart and thus the sensor has a wider working frequency band of 0.2–3 THz; secondly, the refractive index sensitivity of the two resonances achieves 304 GHz/RIU at resonance 1 and 912 GHz/RIU at resonance 2, respectively, which is superior to previous works [24,31], making the sensor easier to identify kinds of substances with similar refractive indices; finally, both resonances can be used for sensing the refractive index between 1 and 2, enabling one to identify a specific substance at multiple frequency bands simultaneously, which can better meet the specificity of the substances to be measured.

### 3.3. Application Analysis

To demonstrate the sensing potential of the proposed dual-band THz sensor, four types of edible oils were used in the simulation experiment: corn oil ($\varepsilon_{corn}=1.33$), peanut oil ($\varepsilon_{peanut}=1.83$), soybean oil ($\varepsilon_{soybean}=2.21$), and canola oil ($\varepsilon_{canola}=2.73$). Since the permittivity ε is proportional to the refractive index n as $\varepsilon =n^{2}$ [17], the reference refractive index ($n_{ref}$) of these kinds of oils were calculated: corn oil ($n_{corn}=1.153$), peanut oil ($n_{peanut}=1.353$), soybean oil ($n_{soybean}=1.486$), and canola oil ($n_{canola}=1.652$). The thickness of the analyte layer was set to 18 μm based on the numerically calculated results of the sensing performance shown in Table 1.

**Figure 6:**
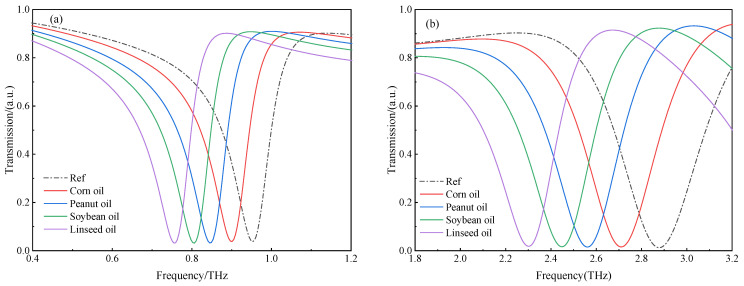
The frequency shift of the transmission spectra of the various types of edible oil at (**a**) $f_{1}$ and (**b**) $f_{2}$.

The ability of the sensor to distinguish different analytes was validated first. Figure 6a,b show the THz transmission spectra of the proposed THz MM sensor at the resonant frequencies $f_{1}$ and $f_{2}$. The resonant peaks of the various types of oil had significantly red-shifted compared with the reference resonant peak of the air (ε=1), and the larger the permittivity of the oil being measured was, the more pronounced the shift of the resonance peak. Table 2 shows the transmission spectra analysis results for various types of edible oil, with air as a reference analyte. The rows of $f_{1}$ and $f_{2}$ are the frequencies of different analytes at resonances 1 and 2 (e.g., the resonant frequencies of corn oil were 0.90 and 2.71 THz, while they were 0.75 and 2.30 THz for canola oil). As a result, it was possible to determine the type of oil being measured based on the resonant frequency of the transmission spectra.

In order to further verify that the proposed sensor can achieve multi-point feature matching at different frequency bands, the refractive indices $n(f_{1})$ and $n(f_{2})$ of each oil sample at different resonant modes were calculated by using Formula (4) according to the resonant peak offset of their transmission spectra, and compared with their standard refractive index $n_{ref}$.

(4) $$n_{\mathrm{oil}}=1+∆n=1+\frac{∆f}{S(f)}$$

where ∆f is the shift of the resonant peak caused by the addition of the edible oil sample, which is shown in rows $∆f_{1}$ and $∆f_{2}$ of Table 2, and ∆n is the variation of the refractive index in comparison to $n_{air}=1$. S(f) represents the refractive index sensitivity, which was previously calculated in Table 1.

As shown in Table 2, the refractive indices of the measured oil, $n(f_{1})$ and $n(f_{2})$, are similar and close to the standard value $n_{ref}$. Take peanut oil as an example: the $n(f_{1})$ and $n(f_{2})$ are 1.368 and 1.354 at mode $f_{1}$ and $f_{2}$, respectively, and the standard refractive index is 1.353. In other words, the characterization results of the transmission spectra in both detection frequency bands can be used to distinguish the types of measured samples, implying that the available discrepancy information obtained by using the proposed dual-band senor for substance detection is doubled when compared with the single-band sensor. Currently, most studies on edible oil spectroscopy detection rely on data from post-processing methods to improve detection accuracy [33,34] but fail to collect more characteristic information during the detection process. In turn, the proposed dual-band terahertz metamaterial sensor can realize multi-point feature matching to achieve high sensitivity and the rapid detection of edible oils, which has a certain reference value for practical applications such as edible oil adulteration detection or gutter oil identification, mainly attributed to the extended detection frequency range and increased number of resonant peaks.

In addition, compared with the spectral analysis results for peanut oil, soybean oil, and canola oil, although the refractive indices of corn oil calculated in both detection bands can be tested with each other, they are still significantly different from the standard refractive indices ($n(f_{1})=1.184$, $n(f_{2})=1.189$, and $n_{ref}=1.153$). This is because the proposed sensor has different detection accuracies for analytes in different refractive index ranges. It can be observed that the analytical results of corn oil with $n_{ref}=1.153$ are not as accurate as those of other edible oils with $n_{ref}\in [1.35,1.65]$. Therefore, further research on tunable sensors that can simultaneously accommodate multiple analytes needs to be carried out based on current work.

## 4. Conclusions

A dual-band high-sensitivity THz MM sensor based on SMSRR was proposed for sensing applications in the frequency range of 0.1–3 THz, with significant resonant peaks in both bands of 0.1–1 THz and 2–3 THz. The SMSRR structure exhibited electromagnetic response with a Q-factor of 7.65 at 0.97 THz with a modulation depth of 96%, and 7.87 at 2.88 THz with a modulation depth of 99%. The sensing performance of the THz MM sensor was numerically simulated by sensing the analytes with various refractive indices for different analyte layer thicknesses. As a result, the best refractive index sensitivities of $S(f_{1})=304$ GHz/RIU and $S(f_{1})=912$ GHz/RIU were achieved with an analyte thickness of 18 μm and with corresponding FOM values of 2.47 and 2.49. By analyzing the terahertz transmission spectra of four edible oils obtained using this metamaterial sensor, it was illustrated that the characterization information of the measured edible oil obtained at the two resonant frequencies basically matched each other, and that different kinds of samples were distinguished according to the shift value of the resonant frequencies. In comparison to most conventional sensors with a single resonant frequency, the proposed sensor achieved multi-point feature matching, allowing one to characterize identical samples at different frequencies, thereby improving the selectivity and sensitivity of detection. Thus, broadening the terahertz detection band and accomplishing multiple resonances are important research directions for terahertz sensing technology. In conclusion, this work establishes a theoretical foundation for the fabrication of a high-sensitivity terahertz sensor, which is of significance for the development of terahertz detection methods.

## Figures and Tables

**Table 1:** Performance of the proposed THz MM sensor with different analyte thicknesses.

Thickness/μm	$S(f_{1})/\mathrm{GHz}\cdot \mathrm{RIU}{}^{-1}$	$S(f_{2})/\mathrm{GHz}\cdot \mathrm{RIU}{}^{-1}$	$FOM(f_{1})/\mathrm{RIU}{}^{-1}$	$FOM(f_{2})/\mathrm{RIU}{}^{-1}$
2	188	573	1.53	1.57
6	271	823	2.20	2.25
10	295	893	2.40	2.44
14	290	898	2.36	2.45
18	304	912	2.47	2.49
22	292	907	2.37	2.48

**Table 2:** Analysis results of the transmission spectra for edible oils detection using the proposed dual-band sensor.

Parameters	Reference Values	Corn Oil	Peanut Oil	Soybean Oil	Canola Oil
ε	1	1.33	1.83	2.21	2.73
$n_{ref}$	1	1.153	1.353	1.486	1.652
$f_{1}$/THz	0.9568	0.9009	0.845	0.802	0.7504
$f_{2}$/THz	2.8832	2.7112	2.5607	2.4489	2.2984
$∆f_{1}$/THz	0	0.0559	0.1118	0.1548	0.2064
$∆f_{2}$/THz	0	0.172	0.3225	0.4343	0.5848
$S(f_{1})$/GHz·RIU^−1^	304				
$S(f_{2})$/GHz·RIU^−1^	912				
$n(f_{1})$	1	1.184	1.368	1.509	1.678
$n(f_{2})$	1	1.189	1.354	1.476	1.641

## Data Availability

The datasets generated and analyzed during the current study are available from the corresponding author on reasonable request.
